# From Nitrate Perception to Environmental Adaptation: Functional Elucidation and Regulatory Networks of the Plant NLP Family

**DOI:** 10.3390/plants15213284

**Published:** 2026-10-24

**Authors:** Hafiz Ishtiaq Ahmad, Ying Liu, Chunrui Chen, Lijie Jia, Huihui Zhu, Jianli Yang

**Affiliations:** 1 College of Resources and Environment, Yunnan Agricultural University, No. 452, Fengyuan Road, Panlong District, Kunming 650201, China; hafizishtiaqahmad786@gmail.com (H.I.A.); 2025110065@stu.ynau.edu.cn (Y.L.); 2021210287@stu.ynau.edu.cn (L.J.); 2 Key Laboratory of Vegetable Biology of Yunnan Province, College of Landscape and Horticulture, Yunnan Agricultural University, No. 452, Fengyuan Road, Panlong District, Kunming 650201, China; chenchunrui@webmail.hzau.edu.cn

**Keywords:** NIN-like protein, nitrate signaling, nitrogen use efficiency, post-translational modification, abiotic stress, symbiotic nitrogen fixation

## Abstract

Nitrogen is a necessary macronutrient that limits plant growth and crop productivity, yet excessive fertilizer application has resulted in serious environmental consequences. The NIN-like protein (NLP) family consists of plant-specific transcription factors that serve as central regulators of nitrate signaling. In 2022, the discovery of NLP7 as an intracellular nitrate receptor represented a major conceptual shift from transcriptional regulation to signal perception. This review systematically summarizes the molecular architecture of NLP proteins, focusing on the conserved RWP-RK DNA-binding domain, the PB1 protein-protein interaction module, and the GAF-like domain associated with the integration of multiple signals. It also examines the species-specific expansion and evolutionary diversification of the NLP family, as well as the multilayered mechanisms that regulate NLP activity, including phosphorylation as a signal-dependent molecular switch, SUMOylation as a negative feedback mechanism, transcriptional regulation of downstream targets, and PB1-mediated protein interaction networks. In addition to nitrate signaling, NLPs serve as central hubs linking nitrogen and carbon metabolism and mediating responses to abiotic stresses including cold, heat, salinity, and iron deficiency. Notably, the NLP7–NIGT1.3 module coordinates a growth–stress trade-off during iron deficiency by modulating nitrate signaling intensity, revealing a previously unrecognized mechanism for balancing stress adaptation with growth maintenance. NLPs also participate in developmental processes, including lateral root formation and symbiotic nodulation. Finally, this review highlights the potential of NLP-based approaches for crop genetic improvement, such as overexpression of key *NLP* genes or CRISPR/Cas-mediated editing, to improve nitrogen use efficiency, stress tolerance, and yield. Collectively, these insights provide a theoretical foundation for developing crop varieties with improved nitrogen-use efficiency and context-dependent stress adaptation, while acknowledging the inherent trade-offs between productivity and resilience.

## 1. Introduction

### 1.1. The Nitrogen Paradox: From Vital Resource to Environmental Liability

Nitrogen is essential for plants, serving as a major component of chlorophyll, nucleic acids, proteins, and various phytohormones, and consequently playing a fundamental role in plant growth, development, and yield formation. To sustain the food supply for a rising global population, nitrogen fertilizer use has increased dramatically in recent decades. However, crops typically utilize only about 30–40% of the nitrogen applied through fertilizers, while a considerable proportion is lost through leaching, surface runoff, and volatilization [1,2]. This inefficiency results in significant economic losses as well as serious environmental issues, including increased greenhouse gas emissions, eutrophication of water bodies, and soil acidification [3,4,5]. Therefore, improving nitrogen use efficiency (NUE) without compromising crop yield has become a major goal of sustainable agriculture worldwide [6]. Achieving this goal requires a better understanding of the mechanisms underlying plant nitrogen uptake, assimilation, and signaling, and the discovery of the NIN-like protein (NLP) family has provided important advances in understanding these processes [7].

### 1.2. From Nodulation Control to Nutrient Sensing: Historical Development and Conceptual Transition in NLP Research

The study of the NLP family reflects a progressive shift in understanding, moving from “specialized function” to “broad relevance”, and from “downstream effector” to “upstream sensor”. Research on this family originated in studies of legume-rhizobium symbiosis. In 1999, the NIN (Nodule Inception) gene was cloned from the model legume *Lotus japonicus* and identified as an essential regulator of nodule organogenesis [8]. Because NIN encodes a protein containing the distinctive RWP-RK domain, it was recognized as an early member of a plant-specific transcription factor family.

The first true NLP genes were discovered in the early 2000s through genomic and phylogenetic analyses. As sequenced plant genomes became available, researchers identified NIN homologs not only in legumes but also in non-nodulating species such as *Arabidopsis thaliana* and rice [9,10,11,12]. These homologs were collectively named NIN-like proteins (NLPs) to distinguish them from the legume-specific NIN. This evolutionary trajectory revealed a striking pattern: while NIN was retained exclusively in legumes for symbiosis, the broader NLP family expanded and diversified across all land plants, suggesting a more general role in nitrogen sensing and signaling.

The initial functional characterization of NLPs in the late 2000s and early 2010s established them as central transcriptional regulators of nitrate signaling. Genetic screens in Arabidopsis identified nlp7 mutants with impaired nitrate responses, and subsequent molecular studies demonstrated that NLP7 and related proteins rapidly accumulate in the nucleus upon nitrate availability, bind promoters of downstream genes such as NRT1.1 and NIA1, activate their transcription, and initiate the Primary Nitrate Response (PNR) [10,13,14]. This period also revealed that NLPs are not merely downstream effectors but master regulators of the nitrate transcriptional network, controlling both nitrate uptake and assimilation.

A major conceptual shift occurred in 2017, when the discovery of the CPK-NLP signaling module demonstrated that NLP7 activity is regulated by calcium-dependent phosphorylation, positioning NLPs closer to the perception step [15]. This understanding changed dramatically in 2022, when Liu et al. revealed that *Arabidopsis* NLP7 itself functions as an intracellular nitrate receptor [16]. Nitrate binds directly to the GAF-like domain of NLP7, triggering a conformational rearrangement that relieves autoinhibition, promotes nucleo-cytoplasmic trafficking, and activates its transcriptional activity [16,17]. This discovery completed the evolutionary and conceptual transition: NLP7 had evolved from a downstream transcriptional mediator into a direct sensor of nitrate availability. Subsequent research has shown that NLP proteins also play important regulatory roles in other crops. For example, NLP4 contributes to nitrogen use efficiency in rice, highlighting both the functional conservation and diversification of the NLP family across plant species [18].

### 1.3. Beyond Nitrogen: NLP as a Central Integrator of Environmental and Developmental Signals

As research in this area has progressed, the functional scope of NLP proteins has expanded considerably. Increasing evidence suggests that NLPs play a crucial role in integrating environmental inputs and developmental pathways [19].

First, NLPs serve as important regulatory nodes connecting nitrogen nutrition with adaptation to abiotic stress. Studies have demonstrated that NLP7 is involved not only in nitrate responses but also in responses to low temperature, high temperature, and salinity. For example, cold exposure rapidly activates the calcium-dependent protein kinase CPK28, which phosphorylates cytoplasmic NLP7 and encourages its translocation into the nucleus, where it activates the expression of cold-responsive genes, including CBF1, CBF2, and CBF3, thus improving freezing tolerance [20]. Similarly, NLP7 contributes to the positive effect of nitrate supply on acquired thermotolerance [21,22]. In addition, heterologous expression of *SsNLP7A* from the halophyte *Suaeda salsa* significantly enhances salt tolerance in tomato, highlighting both the functional plasticity of NLP proteins and their translational potential across species [23]. In contrast, loss of *AtNLP7* function in *Arabidopsis* increases salt tolerance, demonstrating species-dependent functions in salinity responses [24]. Collectively, these results provide a molecular framework for understanding how nitrogen-based nutrition can improve stress resilience.

Second, NLPs are closely involved in the regulation of developmental processes. In addition to their role in nodulation in legumes, NLP proteins strongly influence root system architecture in non-legume species, particularly lateral root development. Nitrate signaling activates *TRYPTOPHAN AMINOTRANSFERASE RELATED 2 (TAR2)* through NLP7, thereby affecting auxin biosynthesis and transport and modulating lateral root initiation and elongation [25]. The identification of the miR156-Squamosa promoter-binding protein-like 9/23 (SPL9/23)-NLP7 regulatory module further connects nitrogen signaling to age-dependent developmental pathways, leaf growth, and plant architecture. This module operates through species-specific regulators, such as AtSPL9–AtNLP7 in *Arabidopsis* and MdSPL23–MdNLP7 in apple [26]. NLPs also cooperate with transcription factors such as teosinte branched1/cycloidea/proliferating cell factor1–20 (TCP20) to regulate root apical meristem activity [27].

Collectively, the NLP family comprises multifunctional transcription factors that integrate signal perception, transcriptional control, developmental transitions, and stress adaptation. These proteins function not only as major coordinators of efficient nitrogen use but also as molecular junctions linking internal nutrient status to external environmental conditions. Although recent reviews have comprehensively summarized NLP functions in nitrogen signaling, symbiosis, and abiotic stress [12], it remains unresolved how a single NLP hub, especially NLP7, converts multiple nutritional and environmental inputs into signal-specific transcriptional outputs, and how this context-dependent regulatory code can be engineered to optimize growth–stress trade-offs rather than to simultaneously maximize productivity and resilience. This review therefore goes beyond a functional catalogue by integrating: (i) domain-level signal perception, particularly direct nitrate sensing by the GAF-like domain; (ii) species-specific expansion and functional diversification of NLP family members; (iii) context-dependent post-translational regulation, contrasting activating CPK-mediated phosphorylation with inhibitory SnRK1-mediated phosphorylation and SUMOylation; and (iv) systems-level crosstalk, including the NLP7–NIGT1.3 growth–stress trade-off and the NLP–NIN symbiotic brake. By synthesizing these layers, we provide a mechanistic framework for understanding NLP-mediated environmental adaptation and highlight key gaps that must be addressed before NLP-based crop engineering can be rationally applied.

## 2. Family Overview: Molecular Architecture, Evolution, and Diversification of NLP Proteins

### 2.1. Structural Characteristics and Functional Domain Organization

**Figure 1:**
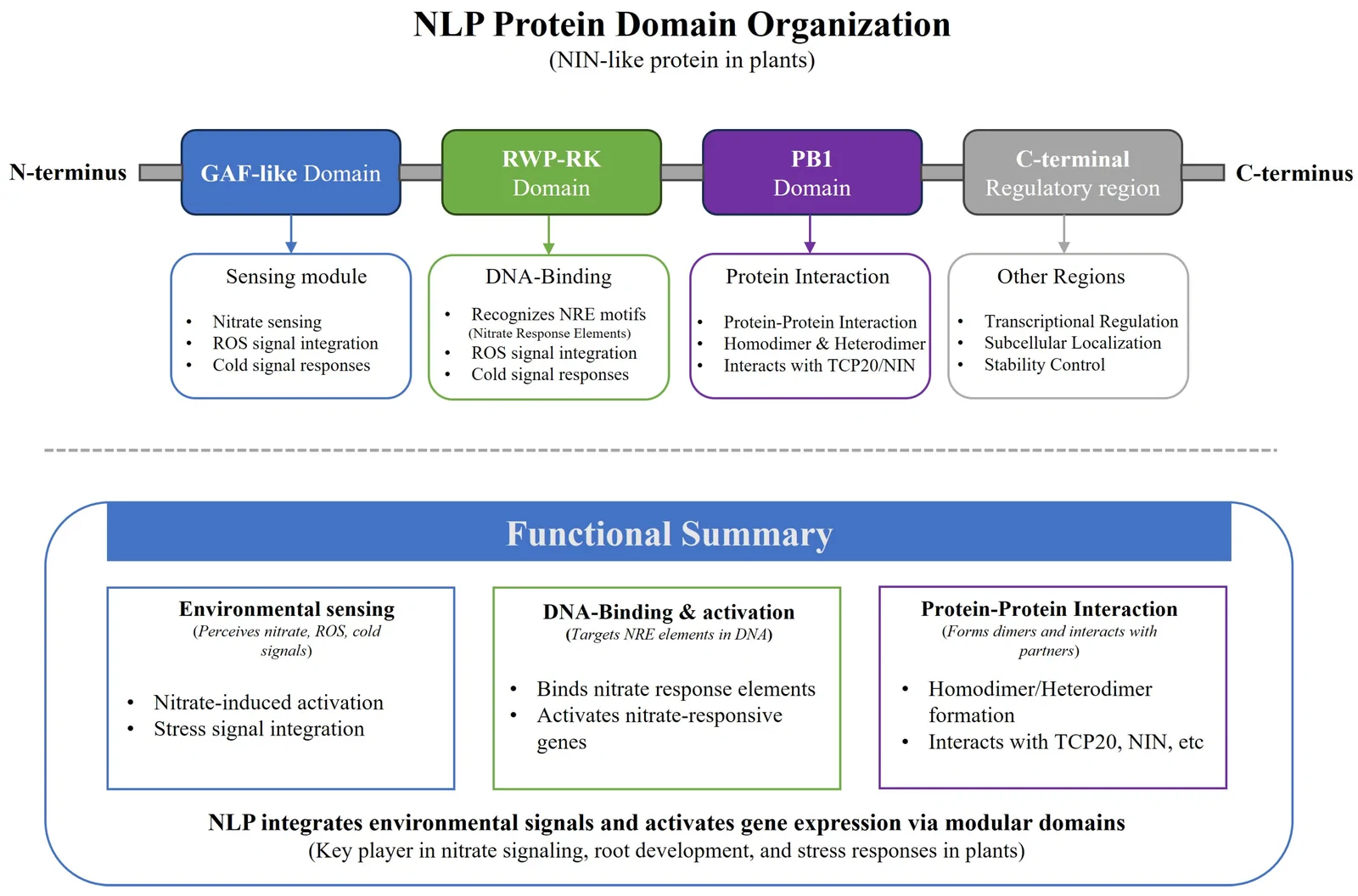
Domain organization and functional modules of NLP proteins. NIN-like proteins (NLPs) exhibit a modular domain architecture spanning from the N-terminus to the C-terminus. The N-terminal GAF-like domain functions as a sensory module that integrates environmental signals, including nitrate availability, reactive oxygen species (ROS), and cold stress. The central RWP-RK domain is responsible for DNA binding, specifically recognizing nitrate-responsive cis-elements (NREs) in target gene promoters and activating transcription. The PB1 domain mediates protein--protein interactions, enabling homo- and heterodimerization as well as interactions with transcriptional regulators such as TCP20 and NIN. The C-terminal region contains additional regulatory elements that contribute to transcriptional control and signal integration. Together, these domains enable NLP proteins to coordinate environmental signal perception with transcriptional regulation of nitrate-responsive genes.

Comparative genomic analyses of diverse plant species, including *Arabidopsis*, rice (*Oryza sativa*), maize (*Zea mays*), cotton (*Gossypium hirsutum*), soybean (*Glycine max*), poplar (*Populus*), apple (*Malus domestica*), tea (*Camellia sinensis*), cucumber (*Cucumis sativus*), and sweet potato (*Ipomoea batatas*), have demonstrated that the NLP family shares a conserved structural framework (Figure 1).

#### 2.1.1. Conserved Core Domains: RWP-RK and PB1

All NLP proteins, regardless of species origin or degree of functional specialization, possess two defining conserved domains.

The RWP-RK domain, which gives the family its name, is approximately 50–60 amino acids in length and contains a highly conserved “RWPxRK” motif. This domain mediates DNA binding by specifically recognizing the NRE located in target gene promoters, thereby enabling activation or repression of downstream transcription [28]. Secondary structure predictions and computational modeling indicate that the RWP-RK domain is predicted to contain two α-helices and an amphipathic leucine zipper, a structural arrangement that may represent a distinctive mode of DNA recognition. Experimental work has confirmed that this domain is indispensable for NRE binding and can function independently in DNA interaction, although its activity is modulated by other regions of the protein [9,11,29,30].

**Figure 2:**
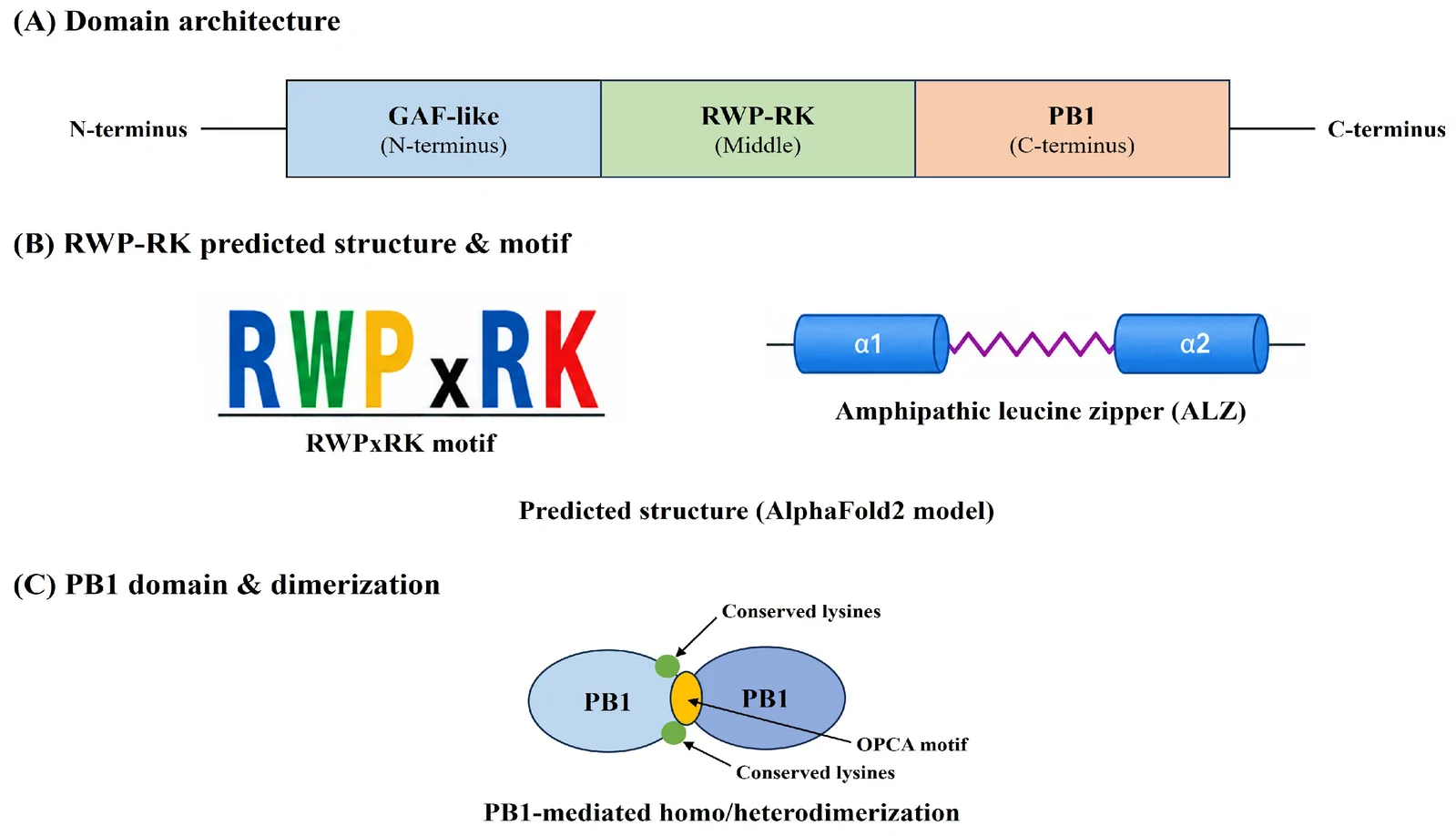
Conserved domains and structural features of NLP proteins. (**A**) Schematic representation of the typical NLP domain architecture, showing the N-terminal GAF-like domain, the central RWP-RK DNA-binding domain, and the C-terminal PB1 domain. (**B**) Predicted structural model of the RWP-RK domain, showing two α-helices (α1 and α2) and an amphipathic leucine zipper (ALZ). The highly conserved “RWPxRK” motif is highlighted. (**C**) Schematic of PB1-mediated homo- and heterodimerization, highlighting the conserved motif.

The PB1 (Phox and Bem1) domain, located at the C-terminus, is approximately 80 amino acids long and serves as a highly conserved protein-protein interaction module [31], (Figure 2). Through this domain, NLP proteins can form homo- and heterodimers with other family members, such as NLP6 and NLP7 [32], and can also associate with unrelated regulatory proteins including TCP20 and, in legumes, NIN [27,33]. These interactions are essential for refining NLP activity, as they allow the formation of higher-order transcriptional complexes that support signal integration and precise regulatory output.

#### 2.1.2. The GAF-like Domain: An Advanced Module for Multi-Signal Integration

Many NLP proteins also contain an N-terminal GAF-like domain, which extends their function from simple nitrate-responsive regulators to complex signal-integration platforms.

In *Arabidopsis*, the N-terminal region of NLP7, featuring a GAF-like domain, is essential for nitrate signaling. Although the GAF-like domain was previously thought to only play a role in the nitrate deficiency-induced nuclear export of NLP7 and the consequent decrease of reactive oxygen species (ROS) upon nitrate exposure [17], direct binding assays have since demonstrated that it is the primary nitrate sensor. Specifically, Liu et al. [16] used microscale thermophoresis (MST) and surface plasmon resonance (SPR) to show that nitrate directly binds to the N-terminal region of NLP7—which contains the GAF-like domain—with a dissociation constant (Kd) in the low-micromolar range. The crystal structure revealed that nitrate binds to a specific pocket within this GAF-like domain, which is structurally similar to the bacterial nitrate sensor NreA. This interaction induces conformational changes that relieve autoinhibition and activate downstream transcriptional programs [16]. Together, these findings indicate that the N-terminal region of NLP7 functions as a multifunctional regulatory hub, with the GAF domain serving as the direct nitrate-binding sensor and neighboring regions contributing to nucleocytoplasmic partitioning and redox-related signaling [16,17].

Recent studies have also suggested a role for this domain in cold signaling. Although CPK28 rapidly phosphorylates NLP7 under cold stress, cold-induced trafficking of NLP7 remains dependent on the GAF-like domain [20]. The specific ligand or molecular trigger involved has not yet been identified. Nevertheless, this finding suggests that the GAF-like domain has functions beyond nitrate sensing alone. Instead, it appears to serve as a flexible integration module through which different environmental signals, including nitrogen status, oxidative signals, and low temperature, converge on NLP proteins.

Thus, the structural organization of NLP proteins can be considered as a modular system consisting of a conserved RWP-RK DNA-binding domain, a PB1 interaction module, and a specialized GAF-like sensory domain. Collectively, these domains enable flexible and coordinated regulation in response to diverse signaling inputs.

### 2.2. Species-Specific Expansion and Lineage-Specific Family Size Variation

The number of NLP genes varies considerably across plant species, reflecting differences in evolutionary history, genome structure, and ecological adaptation (Table 1). Published phylogenetic analyses place conserved NLP members into several major clades and resolve lineage-specific expanded clusters in polyploids and in tea [9,11,34,35,36]. Because the species included here differ in annotation quality and ploidy, we use these published phylogenies as the basis for describing lineage-specific expansion rather than performing a de novo phylogenetic analysis. The species included in Table 1 were selected based on three criteria: (i) availability of a published genome-wide identification of the NLP family, (ii) representation of major agronomic and model plant families, and (iii) inclusion of species with contrasting ploidy levels (diploid, allotetraploid, hexaploid) to examine the relationship between polyploidization and NLP family size. In most diploid plants, NLP family size remains relatively stable, typically in the single digits. For example, *Arabidopsis* contains 9 NLP genes [9], rice 6 [18], maize 9 [37], tomato 6 [38], pepper 7 [39], apple 5 [40], cucumber 3 [41], foxtail millet 7 [42], mungbean 7 [43], and cassava 7 [44]. As reported in published phylogenies [9,11,34,35,36], these conserved members generally fall into several major NLP clades and play fundamental roles in nitrate signaling and developmental regulation.

Notably, some lineages such as Cucurbitaceae (e.g., cucumber and watermelon) possess relatively few NLP genes, suggesting gene loss or suppressed expansion during evolution [41,45]. In contrast, other species exhibit marked NLP family expansion, particularly in polyploid crops such as cotton [46], alfalfa (*Medicago sativa*) [47], peanut (*Arachis hypogaea*) [48], and *Brassica napus* [34,35]. This increase is largely attributable to whole-genome duplication (WGD) followed by partial retention of duplicated copies. However, expansion is not restricted to polyploids. The tea plant, a diploid species, contains 33 NLP genes, indicating that lineage-specific tandem and segmental duplications can also drive expansion [36]. In perennial woody species such as poplar [49] and rhododendron [50], the expanded NLP repertoire may facilitate adaptation to heterogeneous and fluctuating soil environments over long lifespans.

Lineage-specific expansion of the NLP family provides raw material for functional diversification. Some duplicated genes retain core roles in nitrate signaling, whereas others show tissue-specific or stress-related expression specialization [11]. Functional redundancy among paralogs may also confer buffering capacity and enhance the robustness of environmental adaptation. Because these inferences are based on published phylogenies and heterogeneous genome annotations, we refer to this pattern as lineage-specific family size variation rather than proposing a new phylogenetic classification.

Not all expanded members are retained; some lineages have undergone extensive gene loss. For instance, although sweet potato (*Ipomoea batatas*) is hexaploid, its NLP number (7) is lower than that of its diploid relatives *Ipomoea trifida* (9) and *Ipomoea triloba* (12). This observation is consistent with the hypothesis of selective elimination of duplicate genes after polyploidization to maintain genome stability and functional balance [51]. However, lower gene counts alone cannot definitively establish selective gene loss, as differences in genome annotation or lineage-specific duplications in the diploid relatives could also contribute to this pattern.

**Table 1:** Comparison of NLP family member numbers in different plant species.

Species	Family/Genus	Genome Characteristics	Chromosome Number	NLP Number	References
*Arabidopsis thaliana*	Brassicaceae	Diploid	2n = 10	9	[9]
*Oryza sativa*	Poaceae	Diploid	2n = 24	6	[18]
*Zea mays*	Poaceae	Diploid	2n = 20	9	[37]
*Solanum lycopersicum*	Solanaceae	Diploid	2n = 24	6	[38]
*Capsicum annuum*	Solanaceae	Diploid	2n = 24	7	[39]
*Malus domestica*	Rosaceae	Diploid	2n = 34	5	[40]
*Cucumis sativus*	Cucurbitaceae	Diploid	2n = 14	3	[41]
*Citrullus lanatus*	Cucurbitaceae	Diploid	2n = 22	3	[45]
*Setaria italica*	Poaceae	Diploid	2n = 18	7	[42]
*Vigna radiata*	Fabaceae	Diploid	2n = 22	7	[43]
*Manihot esculenta*	Euphorbiaceae	Diploid	2n = 36	7	[44]
*Camellia sinensis*	Theaceae	Diploid	2n = 30	33	[36]
*Populus trichocarpa*	Salicaceae	Diploid	2n = 38	14	[49]
*Rhododendron henanense*	Ericaceae	Diploid	2n = 26	11	[50]
*Medicago sativa*	Fabaceae	Autotetraploid	2n = 32	53	[47]
*Gossypium hirsutum*	Malvaceae	Allotetraploid	2n = 52	>100	[46]
*Arachis hypogaea*	Fabaceae	Allotetraploid	2n = 40	16	[48]
*Brassica napus*	Cruciferae	Allotetra-ploid	2n = 38	31	[34]
*Brassica rapa*	Cruciferae	Diploid	2n = 20	17	[35]
*Brassica oleracea*	Cruciferae	Diploid	2n = 18	16	[35]
*Brassica napus*	Cruciferae	Allotetra-ploid	2n = 38	32	[35]
*Ipomoea trifida*	Convolvulaceae	Diploid	2n = 30	9	[51]
*Ipomoea triloba*	Convolvulaceae	Diploid	2n = 30	12	[51]
*Ipomoea batatas*	Convolvulaceae	Hexaploid	2n = 90	7	[51]

## 3. Core Mechanisms: The Multilayered Regulatory System Governed by NLP Proteins

The central role of NLP proteins in nitrate signaling and environmental adaptation depends on a dynamic regulatory framework that spans signal perception, post-translational regulation, transcriptional control, and protein interaction.

### 3.1. Nitrate Sensing: A Closed Signaling Circuit from Membrane Receptor to Nuclear Receptor

Plants do not rely on a single mechanism to detect nitrate availability. Instead, they have evolved a dual-sensing system that ensures reliable recognition and transmission of nitrate signals.

At the plasma membrane, NRT1.1 functions as the first identified nitrate transceptor, combining transport and sensing functions [52]. Under low nitrate conditions, phosphorylation converts it into a high-affinity transporter, whereas under high nitrate, dephosphorylation enables low-affinity transport. Crucially, NRT1.1 also senses extracellular nitrate fluctuations and initiates downstream signaling through conformational changes, thereby serving as the primary external nitrate sentinel.

Inside the cell, NLP7 acts as an intracellular nitrate receptor [16]. Once nitrate enters the cell through NRT1.1 or related transporters, it directly binds the GAF-like domain of NLP7. This interaction induces a conformational shift that relieves autoinhibition and promotes accumulation of NLP7 in the nucleus. There, NLP7 activates transcriptional reprogramming associated with the Primary Nitrate Response.

Together, NRT1.1 and NLP7 form a complete extracellular perception-intracellular decoding system. NRT1.1 monitors soil nitrate availability and conveys that information inward, whereas NLP7 serves as the internal sensor-executor that converts nitrate presence into coordinated changes in gene expression. This system constitutes a closed circuit because NLP7 directly promotes the transcription of *NRT1.1* (and other nitrate transporters/assimilatory genes) as part of the Primary Nitrate Response. This transcriptional feedback edge modulates the abundance of the membrane sensor, allowing the plant to dynamically adjust its nitrate uptake capacity in response to intracellular nitrogen status.

### 3.2. Post-Translational Control: Opposing Roles of Phosphorylation and SUMOylation

NLP activity is highly dynamic and subject to tight post-translational control. Among the most important regulatory mechanisms are phosphorylation and SUMOylation, which together operate as an activating and restraining system that determines the timing, subcellular localization, and strength of NLP action. Importantly, phosphorylation is not a uniformly activating signal. Its functional outcome depends on the specific kinase, the targeted residue, and the cellular context.

#### 3.2.1. Phosphorylation as a Signal-Specific Molecular Switch

In nitrate signaling, nuclear-localized calcium-dependent protein kinases CPK10, CPK30, and CPK32 phosphorylate NLP7 at Ser205. This modification promotes its retention in the nucleus, thereby sustaining activation of genes involved in nitrogen metabolism, including *NRT1.1* and *NIA1* [15]. Under cold stress, the plasma membrane-associated kinase CPK28 is rapidly activated and phosphorylates NLP7 at multiple serine/threonine residues in the cytoplasm, including Ser783, Ser793, Ser807, and Ser808 [20]. This triggers nuclear import, allowing NLP7 to activate cold-responsive genes and improve freezing tolerance. Heat stress also induces nuclear accumulation of NLP7, which subsequently activates *HSFA2* and other heat-responsive transcription factors [21,22]. Importantly, nitrate availability enhances this NLP7-dependent heat response, providing a direct molecular basis for the beneficial effect of nitrogen nutrition on thermotolerance. However, direct phosphorylation of NLP7 under heat stress has not yet been experimentally demonstrated. While the responsible kinase(s) and modification sites remain unknown, the involvement of phosphorylation in this pathway is currently an untested possibility.

In contrast to the activating phosphorylation, NLP7 is also subject to inhibitory phosphorylation by the evolutionarily conserved energy sensor SnRK1. Under carbon deficiency or nitrate depletion, the SnRK1 catalytic subunit KIN10 is activated and directly phosphorylates NLP7 at Ser125 and Ser306. This modification promotes the cytoplasmic localization and degradation of NLP7, thereby repressing nitrate signaling and the downstream Primary Nitrate Response. Mutation of KIN10 partially suppresses the inhibitory effects of carbon deficiency on nitrate-mediated plant growth, confirming that this phosphorylation event functions as a negative regulatory edge in the NLP7 signaling network [53].

#### 3.2.2. SUMOylation as a Negative Regulatory Brake

In addition to inhibitory phosphorylation by SnRK1, NLP activity is further restrained by SUMOylation. While phosphorylation can both activate and inhibit NLP function, SUMOylation appears to primarily act as a negative regulatory brake. Recent studies reveal that nitrate signaling promotes not only NLP phosphorylation but also NLP SUMOylation [54]. SUMOylation involves the covalent attachment of a Small Ubiquitin-like Modifier (SUMO) to lysine residues on target proteins. In NLP proteins, nitrate-induced SUMOylation appears to occur during the later phases of activation and serves as part of a negative feedback mechanism.

In particular, SUMOylation may decrease the transcriptional activity of NLP or promote its export from the nucleus, thereby attenuating nitrate-induced responses. This establishes a regulatory circuit wherein nitrate activates NLP, NLP induces downstream genes, and this activation leads to NLP SUMOylation, which suppresses further activity. Such a coordinated accelerator–brake mechanism ensures that nitrate responses are rapid but appropriately limited, thereby preventing detrimental overactivation or unnecessary energy expenditure. This regulatory mechanism appears to be conserved across different species and may represent an important component of the NLP regulatory network.

Taken together, these observations establish NLP7 as a genuine multi-signal integration node. By being phosphorylated at distinct sites by different kinases in different subcellular compartments, NLP7 can discriminate among upstream cues such as nitrate, cold, heat, and carbon status. Critically, the functional consequence of phosphorylation is context-dependent: CPK-mediated phosphorylation promotes nuclear retention and activation, whereas SnRK1-mediated phosphorylation promotes cytoplasmic degradation and inhibition. This dual phosphorylation code allows NLP7 to function as both an activator and a repressor of nitrate signaling, depending on the prevailing metabolic and environmental conditions (Table 2).

### 3.3. Transcriptional Reprogramming: Identification and Functional Validation of Downstream Targets

Upon activation and accumulation in the nucleus, NLP proteins trigger broad transcriptional cascades by binding to the promoters of target genes and reprogramming cellular responses.

Genome-wide approaches, including ChIP-seq, have revealed several direct NLP targets. Alongside genes involved in nitrogen metabolism, such as nitrate transporters and reductases, NLPs also activate genes encoding secondary transcription factors, which further transmit and diversify the response. For instance, NLP7 directly binds to the promoter of WRKY20, thereby stimulating its expression [55]. In response to heat stress, NLP7 also binds to the promoters of *HSFA2*, *HSFA3*, and *BOBBER1* (*BOB1*), thus activating heat-responsive transcriptional programs [21,22].

These secondary factors contribute to broader regulatory cascades. In the NLP7-WRKY20-NRT pathway, WRKY20 directly activates *NRT1.1* and *NRT2.1*, thereby promoting nitrate uptake and transport [55]. In the NLP7-HSFA2-HEAT SHOCK PROTEIN (HSP) pathway, NLP7-induced HSFA2 activates a large set of heat shock protein genes, including HSP18.2, HSP22, and *HEAT STRESS ASSOCIATED 32* (*HSA32*). These proteins serve as molecular chaperones and help maintain protein homeostasis under heat stress [22].

Additionally, NLPs link nutrient signaling with developmental regulation. The miR156-SPL9/23-NLP7 module connects nitrogen availability with root development [26]. In *Arabidopsis*, AtSPL9 represses AtNLP7, while in apple, MdSPL23 represses MdNLP7. Under nitrogen-rich environments, SPL transcription factors repress *NLP7* expression, restricting lateral root development. Under nitrogen deficiency, this repression is reduced, resulting in increased NLP7 activity and improved lateral root development to aid in nutrient acquisition.

Notably, NLP-centered networks also contain negative regulatory pathways. One example is the antagonistic relationship between WRKY20 and NITRATE-INDUCIBLE GARP-TYPE TRANSCRIPTIONAL REPRESSOR1.4 (NIGT1.4) at the NRT2.1 promoter [55]. WRKY20 activates NRT2.1, whereas NIGT1.4 represses it. Their combined action allows NRT2.1 expression to increase rapidly following nitrate stimulation and subsequently decline to a moderate level, thus preventing excessive nitrate uptake.

### 3.4. Protein Interaction Networks: PB1-Mediated Functional Cooperation

The function of NLP proteins does not rely solely on their intrinsic properties but also on their ability to interact with other proteins. The PB1 domain plays a central role in this process.

NLP proteins are capable of forming both homo- and heterodimers. For example, *Arabidopsis* NLP6 and NLP7 interact through their PB1 domains to form heterodimers that jointly regulate nitrate-responsive genes [32]. This oligomerization may affect the specificity of DNA-binding and the efficacy of transcription.

The PB1 domain also facilitates interactions with non-NLP proteins, enabling integration with other signaling pathways. NLP7 interacts with TCP20, and this interaction is required for regulating root apical meristem activity during nitrogen starvation [27]. In legumes, NLP proteins such as MtNLP1 interact with NIN, establishing the mechanistic basis for nitrate-mediated inhibition of nodulation [12,33].

**Figure 3:**
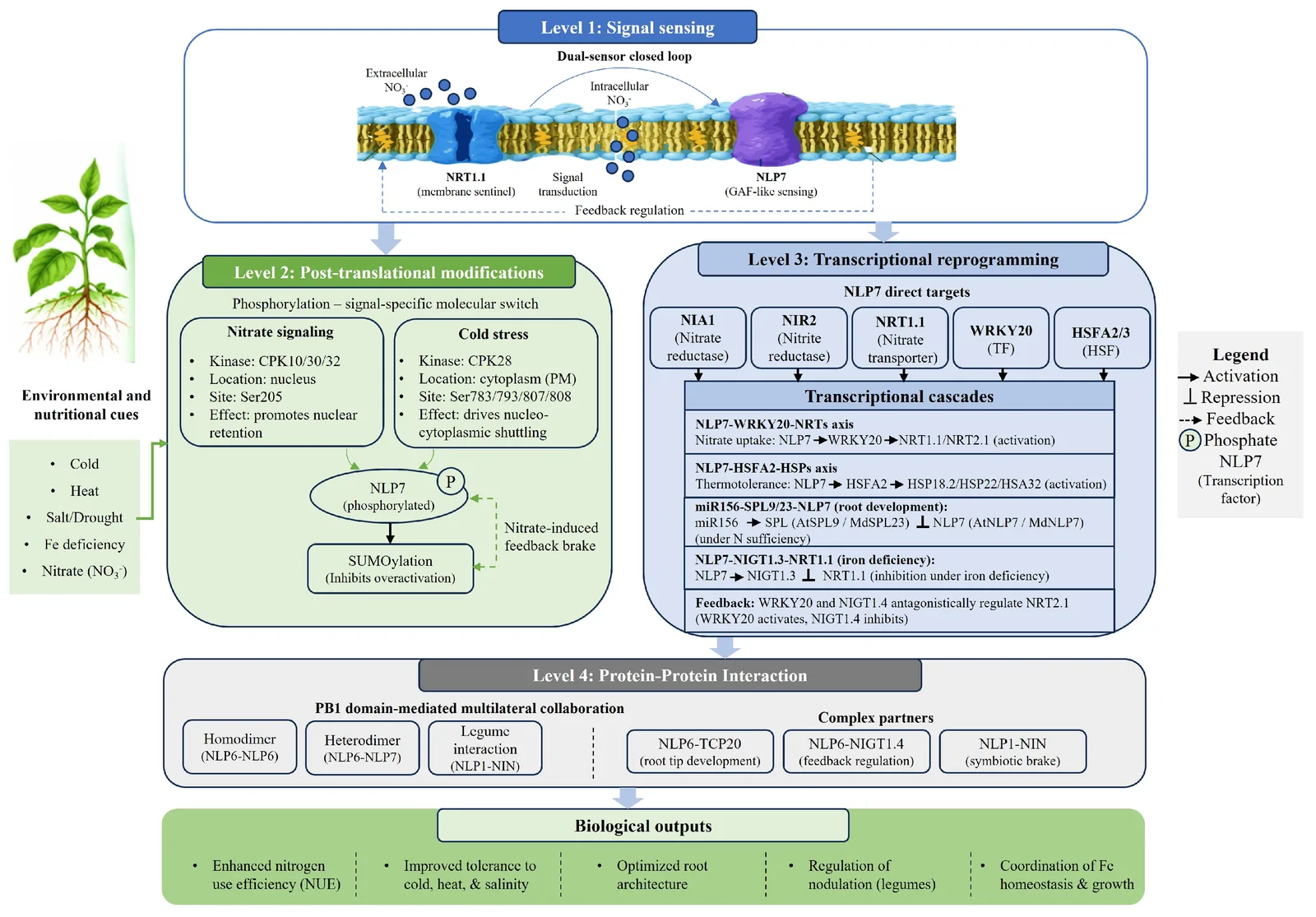
NLP-mediated multilevel regulatory network integrating nitrate signaling and environmental cues. The network is organized across four interconnected layers: Level 1—Signal Sensing: A dual-sensor system detects nitrate at both extracellular and intracellular levels. The transceptor NRT1.1 functions as a membrane-localized sentinel sensing extracellular NO_3_^−^ and initiating signal transduction. Intracellular nitrate is perceived by the GAF-like domain of NLP7, a transcription factor that undergoes conformational changes upon nitrate binding to relieve autoinhibition and promote its activation. Level 2—Post-Translational Modifications: NLP7 activity is fine-tuned by two opposing modifications. Phosphorylation as a signal-specific “molecular switch”: Under nitrate signaling, nuclear-localized CPK10/30/32 phosphorylate NLP7 at Ser205, promoting its nuclear retention and sustained activation of nitrogen metabolism genes. Under cold stress, plasma membrane-associated CPK28 phosphorylates NLP7 at Ser783/793/807/808, driving nucleocytoplasmic shuttling and activation of cold-responsive genes. SUMOylation as a negative “molecular brake”: Nitrate-induced SUMOylation of NLP7 at specific lysine residues attenuates its activity, preventing overactivation and constituting a negative feedback loop. Level 3—Transcriptional Reprogramming: Activated NLP7 regulates diverse downstream targets and transcriptional cascades. (Direct targets): NLP7 directly activates nitrate assimilation genes (NIA1, NIR1), nitrate transporters (NRT1.1), and secondary transcription factors (WRKY20, HSFA2/3). (Transcriptional cascades): The NLP7–WRKY20–NRTs axis enhances nitrate uptake; the NLP7–HSFA2–HSPs axis promotes thermotolerance; the NLP7-NIGT1.3-NRT1.1 axis regulates iron deficiency responses; the miR156-SPL9/23 module (AtSPL9–AtNLP7 in *Arabidopsis* and MdSPL23–MdNLP7 in apple) governs lateral root development. (Feedback inhibition): Antagonistic control of NRT2.1 expression by WRKY20 (activation) and NIGT1.4 (repression) achieves precise signal balance. Level 4—Protein–Protein Interactions: The PB1 domain of NLP7 mediates homo- and heterodimerization (NLP6–NLP7) as well as interactions with multiple partners, including TCP20 (root meristem development), NIGT1.4 (feedback regulation), and NIN (suppression of legume nodulation), thereby integrating developmental and symbiotic signaling pathways. Together, this multilevel architecture enables NLP7 to serve as a central hub integrating nitrate nutrition with diverse environmental cues (cold, heat, salt, iron deficiency) and developmental programs, coordinating plant growth, stress adaptation, and resource allocation.

Collectively, these findings indicate that NLP functions through a complex multilayered regulatory framework, involving signal perception, post-translational modifications (phosphorylation and SUMOylation), transcriptional regulation, and protein-protein interactions. Through these mechanisms, NLP proteins can integrate nitrate signaling with environmental responses, including cold, heat, and salt stress, as well as developmental processes. This places NLP at a central position for coordinating plant growth and stress adaptation (Figure 3).

## 4. Systems-Level Integration: NLP as a Hub in Plant Development and Stress Adaptation

**Figure 4:**
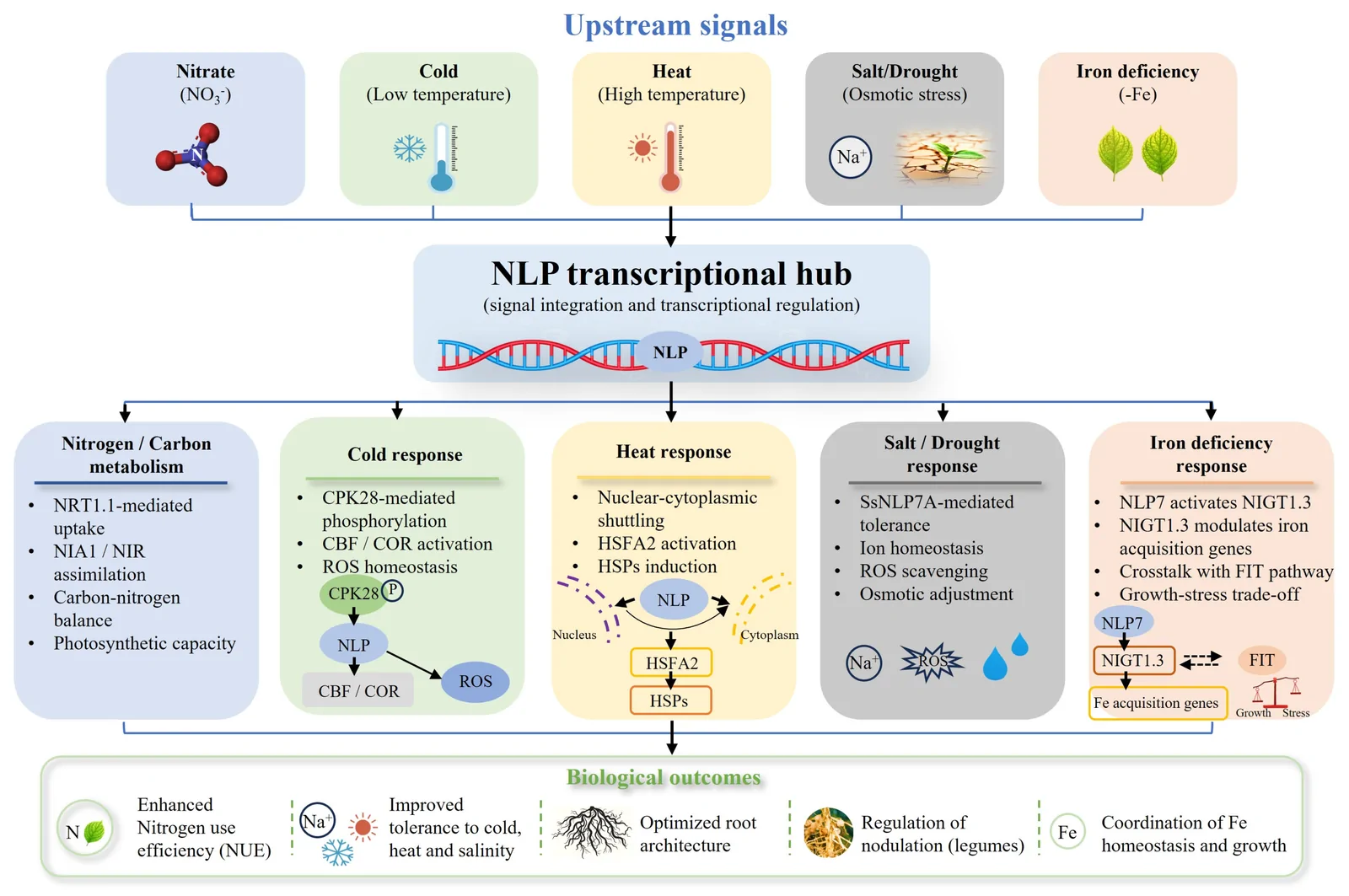
NLP transcription factors function as a central transcriptional hub integrating nitrate signaling with multiple abiotic stress responses. NIN-like proteins (NLPs) coordinate diverse environmental and nutritional cues, including nitrate (NO_3_^−^), cold, heat, salt/drought, and iron deficiency, to regulate plant growth and stress adaptation. Upon perception of these upstream signals, NLPs integrate signaling information through transcriptional regulation and post-translational modulation. Downstream, NLPs regulate five major biological processes: (i) nitrogen and carbon metabolism by promoting nitrate uptake (e.g., *NRT1.1*), nitrate assimilation (*NIA1/NIR*), carbon–nitrogen balance, and photosynthetic capacity; (ii) cold stress responses through CPK28-mediated phosphorylation, activation of the CBF/COR pathway, and maintenance of reactive oxygen species (ROS) homeostasis; (iii) heat stress responses via nucleocytoplasmic shuttling, activation of *HSFA2*, and induction of heat shock proteins (HSPs); (iv) salt and drought tolerance by regulating ion homeostasis, ROS scavenging, and osmotic adjustment, exemplified by the positive role of *SsNLP7A*; and (v) iron deficiency responses through the *NLP7–NIGT1.3* regulatory module, which coordinates iron acquisition, interacts with the FIT-dependent pathway, and balances growth with nutrient stress adaptation. Collectively, these regulatory networks contribute to enhanced nitrogen use efficiency (NUE), improved tolerance to multiple abiotic stresses, optimized root system architecture, regulation of nodulation in legumes, and coordinated maintenance of iron homeostasis and plant growth.

With continued study, it has become clear that NLP proteins are not limited to regulating nitrogen metabolism. Rather, they function as hubs that integrate primary metabolism, development, and environmental stress responses (Figure 4).

### 4.1. NLP and the Coordination of Nitrogen and Carbon Metabolism

Plant growth and yield depend on the coordinated operation of nitrogen and carbon metabolism, and NLP proteins appear to occupy a strategic bridging position in this process.

In *Arabidopsis*, overexpression of *AtNLP7* not only enhances nitrate uptake and assimilation but also improves photosynthetic efficiency and carbon fixation, resulting in increased biomass under both nitrogen-sufficient and nitrogen-deficient conditions [56]. These findings first suggested that NLP7 could act as a node coordinating nitrogen and carbon metabolism.

This concept has been validated in crops. Field studies in cotton expressing *AtNLP7* showed that transgenic plants accumulated greater total nitrogen and carbon content than wild-type controls [57]. These plants also exhibited elevated chlorophyll content, more vigorous vegetative growth, and significantly improved yield and NUE across multiple fertilization regimes, especially under low to moderate nitrogen supply. This demonstrates that manipulating NLP7 expression can enhance productivity by promoting coordinated nitrogen and carbon accumulation.

### 4.2. NLP as an Integration Hub for Abiotic Stress Responses

- Cold Stress

The CPK28-NLP7 module defines a complete pathway from cold perception to transcriptional adaptation [20]. Cold stress activates calcium signaling, which in turn activates CPK28 at the plasma membrane. Activated CPK28 phosphorylates NLP7 in the cytoplasm, triggering its nuclear import. Nuclear NLP7 then activates CBF1–CBF3 and COR genes, thereby enhancing freezing tolerance.

- Heat Stress

Appropriate nitrate nutrition enhances acquired thermotolerance through an NLP7-dependent mechanism [21,22]. Under heat stress, NLP7 accumulates in the nucleus and directly activates HSFA2, which induces heat shock proteins (HSPs), which function as molecular chaperones. This defines a molecular axis linking nutritional status, NLP7 activity, and heat adaptation.

- Salt Stress

NLP7 function in salinity responses appears to be species-dependent, which is itself informative for its role as an integration hub. In *Arabidopsis*, loss of *NLP7* function enhances salt tolerance, suggesting that AtNLP7 can act as a negative regulator in this context [24]. In contrast, overexpression of *SsNLP7A* from the halophyte *Suaeda salsa* improves salt tolerance in tomato [23]. Transgenic plants show reduced Na^+^ accumulation, better K^+^ homeostasis, greater accumulation of osmoprotectants such as proline and soluble sugars, higher antioxidant enzyme activities, and improved photosynthetic performance under salt stress. This striking divergence indicates that NLP homologs from halophytes may have evolved specialized positive roles in stress adaptation and therefore represent valuable breeding resources. Importantly, the species-dependent direction of the salt response does not undermine the hub model; rather, it demonstrates that the hub’s output is context-dependent, consistent with the dual-function phosphorylation switch described in Section 3.2.1.

- Drought and ABA Signaling

Transcriptome analyses in species such as alfalfa and cucumber indicate that multiple NLP genes are responsive to drought simulation and exogenous abscisic acid (ABA) treatment [41,47]. Although the underlying mechanisms remain unresolved, these findings suggest that NLP family members also participate in ABA-associated drought responses. While this evidence is correlative rather than mechanistic, it is consistent with the broader pattern of NLP7-mediated signal integration described above. Future studies identifying the upstream kinases and downstream targets in drought signaling will be necessary to determine whether NLP7 functions as a hub in this context as well.

- Iron Deficiency Response

Recent evidence has also implicated NLP7 in iron (Fe) deficiency signaling. Under Fe deficiency, nitrate triggers NLP7 nuclear accumulation, which in turn directly activates the transcriptional repressor *NIGT1.3*. NIGT1.3 subsequently represses the nitrate transporter *NRT1.1*, forming a feedback loop that balances Fe deficiency responses with plant growth. Loss of NLP7 function attenuates Fe deficiency symptoms but also reduces growth, revealing a trade-off between stress adaptation and development [58]. This finding expands NLP7’s known function beyond nitrate sensing to include micronutrient homeostasis and growth regulation, providing a molecular explanation for the long-observed phenomenon that nitrate fertilization exacerbates Fe deficiency chlorosis in crops. Notably, this growth-stress trade-off echoes the “symbiotic brake” mechanism observed in legumes (Section 4.3), suggesting that NLP-dependent resource allocation strategies may represent a conserved principle across different physiological contexts.

Across these stress contexts, NLP7 receives distinct signals through different upstream kinases and produces signal-specific outputs: CBF1–CBF3 under cold stress, HSFA2–HSPs under heat stress, and NIGT1.3 under iron deficiency. This context-dependent target selection demonstrates that NLP7 integrates stress signals at the level of the node, not by activating a single shared effector. The salt response further shows that hub output is species-dependent. Together, these features—convergent upstream regulation, context-dependent downstream outputs, and growth–stress coordination—justify the characterization of NLP7 as an integration hub for abiotic stress responses. This hub function depends on the downstream target genes (Table 3) alongside a broader network of interacting partners (Table 4) alongside the downstream target genes (Table 3). To systematically visualize the protein partnerships of NLP7, we constructed a STRING-based interaction network using *Arabidopsis thaliana* as the reference organism (Figure S1). The network substantiates the central role of NLP7 in integrating nitrate signaling with stress and developmental pathways.

### 4.3. NLP-Mediated Suppression of Symbiotic Nodulation: A Resource Allocation Strategy

Similar to the growth-stress trade-off observed under Fe deficiency, NLP proteins in legumes regulate resource allocation through their role in balancing the energetic costs and benefits of symbiotic nitrogen fixation.

When nitrogen is abundant in the soil, legumes restrict the formation of additional nodules to conserve resources. This “symbiotic brake” is mediated in part by NLP proteins. In *Medicago truncatula*, increased nitrate availability promotes nuclear accumulation of MtNLP1 [12,33]. Upon entering the nucleus, MtNLP1 interacts directly with MtNIN through its PB1 domain. This interaction obstructs MtNIN-dependent activation of nodulation-related genes, including *cytokinin response 1* (*CRE1*), thereby restricting nodulation.

Although current evidence primarily highlights the NLP-NIN interaction, it is conceivable that post-translational mechanisms such as SUMOylation also play a role in this process. Nitrate-induced SUMOylation may influence NLP1 localization, stability, or affinity for NIN, thereby affecting the strength and duration of nodulation suppression.

## 5. Prospects for Application: From Mechanistic Insight to Crop Improvement

The long-term significance of NLP research lies in its application to agriculture. Current mechanistic knowledge suggests several promising breeding strategies for improving nitrogen-use efficiency and context-dependent stress adaptation, while recognizing the inherent trade-offs between productivity and resilience. In this review, the term “integration” is used in a biological sense: it refers to the convergence of multiple nutritional and environmental signals on NLP proteins, particularly NLP7, and the context-dependent selection of downstream transcriptional programs. It does not refer to a synthetic gene circuit. Engineering approaches that exploit this natural integration function are discussed in Section 5.1 and Section 5.2.

### 5.1. Targets for Genetic Engineering and Molecular Breeding

One direct approach is to manipulate the expression of key NLP genes in crops, though the optimal strategy is likely to be species- and context-dependent. Field studies have shown that overexpression of *AtNLP7* in cotton can improve both yield and NUE [57]. Similarly, overexpression of *OsNLP1*, *OsNLP3*, or *OsNLP4* in rice has been reported to enhance NUE and grain production [18,61,62]. Importantly, these improvements in yield and NUE have been documented under specific nitrogen regimes and do not imply simultaneous enhancement of broad-spectrum stress resilience. The trade-off between productivity and stress adaptation must be evaluated empirically for each target environment. However, NLP7 does not act as a universal positive regulator. For example, in *Arabidopsis*, loss of NLP7 function enhances salt tolerance, indicating that AtNLP7 can act as a negative regulator in specific stress contexts. Therefore, genetic engineering strategies must carefully consider the target crop, the desired trait, and the potential trade-offs between growth and stress tolerance. These findings support the potential application of NLP-based strategies, but they also highlight the need for precise, context-specific manipulation rather than simple overexpression.

Beyond conventional overexpression, the ability of NLP proteins to integrate multiple signals enables more advanced engineering approaches. For example, introducing favorable alleles such as *SsNLP7A* from halophytes into cereal crops could improve both salt tolerance and NUE [23]. Conversely, CRISPR/Cas-based modification of endogenous *NLP* genes could be used to produce phosphomimetic variants at specific activating residues (e.g., Ser205), potentially mimicking the activated state and enhancing baseline NLP activity without requiring transgene introduction. However, because phosphorylation can either activate or inhibit NLP7 depending on the targeted residue (e.g., inhibitory phosphorylation at Ser125/Ser306 by SnRK1), this strategy must be viewed as a site-dependent engineering hypothesis. Functional validation of specific phosphomimetic lines will be required to determine whether such edits enhance nutrient use efficiency or stress tolerance.

Building on the mechanistic understanding of the “symbiotic brake” (Section 4.3), an alternative strategy for legumes could involve disrupting the inhibitory NLP functions that contribute to nodulation suppression. Editing SUMOylation sites or other regulatory regions of genes such as MtNLP1 may reduce the nitrate-induced “symbiotic brake”, facilitating more sustained nodulation and biological nitrogen fixation even under conditions of high soil nitrogen availability.

### 5.2. Synthetic Biology and Precision Engineering of NLP Regulatory Nodes

The natural signal-integration function of NLPs also provides a conceptual template for synthetic biology. Rather than simply overexpressing NLP genes, future engineering could exploit synthetic promoters, synthetic gene circuits, modular gene switches, and RNA-based control elements to fine-tune NLP activity in a dose- and context-dependent manner. At the promoter level, synthetic promoters containing tandem NREs could be used to drive downstream genes specifically in response to nitrate, thereby uncoupling nitrate signaling from endogenous feedback regulation. At the circuit level, NLP7 could serve as a sensor module in synthetic gene circuits that couple nitrate perception to defined output genes, allowing programmable transcriptional responses. At the protein level, CRISPR/Cas-based base editing or prime editing could introduce phosphomimetic or phospho-dead variants at specific NLP7 residues, such as the activating site Ser205 or the inhibitory sites Ser125/Ser306, to adjust the balance between nuclear retention and cytoplasmic degradation. Modular gene switches could be created by fusing NLP7 to chemically inducible or light-controlled domains, enabling external control of its transcriptional activity. At the post-transcriptional level, nitrate-responsive riboswitches or upstream open reading frames could be engineered into NLP7 transcripts to control translation. These strategies remain largely hypothetical and will require functional validation, particularly because NLP7 can act as either a positive or negative regulator depending on the stress context and species. Their value will therefore lie in optimizing growth–stress trade-offs rather than maximizing all traits simultaneously.

## 6. Conclusions

Research over the past decade has established the NLP family as a central molecular hub that integrates nutrient cues, environmental stress signals, and developmental programs. NLP function depends on a sophisticated regulatory architecture that includes direct nitrate sensing, post-translational control through phosphorylation and SUMOylation, transcriptional cascades involving secondary regulators, and extensive protein interaction networks mediated by the PB1 domain.

A deeper understanding of NLP biology has greatly advanced our knowledge of how plants perceive and adapt to fluctuating environments. At the same time, it has generated valuable conceptual and genetic resources for sustainable agriculture. By combining mechanistic insight with modern genome editing and breeding technologies, targeted manipulation of NLP family members holds promise for developing crop varieties with improved nitrogen-use efficiency and context-dependent stress adaptation. Because productivity and resilience often trade off, future efforts should focus on optimizing these trade-offs for specific environments rather than maximizing all traits simultaneously, thereby contributing to sustainable agriculture and global food security.

## Figures and Tables

**Table 2:** Key post-translational modification sites and functions of NLP proteins.

Modification Type	Signal/Stimulus	Kinase/Enzyme	Modification Site	Subcellular Localization	Functional Effect	References
Phosphorylation	Nitrate	CPK10/30/32	Ser205	Nucleus	Promotes nuclear retention, sustained activation of nitrogen metabolism genes	[15]
Phosphorylation	Cold stress	CPK28	Ser783, Ser793, Ser807, Ser808	Cytoplasm	Drives nucleocytoplasmic shuttling, activates cold-responsive genes	[20]
Putative	Heat stress	Unknown	To be identified	Cytoplasm/Nucleus	Drives nucleocytoplasmic shuttling, activates heat-responsive genes	[21,22]
Phosphory-lation	Carbon deficiency/Nitrate depletion	SnRK1 (KIN10)	Ser125, Ser306	Cytoplasm	Promotes degradation; inhibits nitrate signaling	[53]
SUMOylation	Nitrate	SUMO E3 ligase	Specific lysine residues	Nucleus	Negative feedback inhibition, prevents overactivation	[54]

**Table 3:** Representative downstream targets of NLP and their functions.

Target Gene Category	Gene Name	Function	Regulatory Mode	References
Nitrate transporter	*NRT1.1*	Low/high-affinity nitrate transport	Activation	[55]
Nitrate transporter	*NRT2.1*	High-affinity nitrate transport	Activation (antagonistic with NIGT1.4)	[13]
Nitrate assimilation enzyme	*NIA1*	Nitrate reductase	Activation	[13]
Nitrate assimilation enzyme	*NIR1*	Nitrite reductase	Activation	[10]
Secondary transcription factor	*WRKY20*	Transcription factor regulating nitrate transport	Activation	[55]
Secondary transcription factor	*HSFA2*	Heat shock transcription factor, regulates heat stress response	Activation	[21,22]
Secondary transcription factor	*HSFA3*	Heat shock transcription factor, regulates heat stress response	Activation	[21]
Heat shock protein	*HSP18.2*, *HSP22*, *HSA32*	Molecular chaperones, maintain protein homeostasis	Activation (via HSFA2)	[22]
Nitrate/Fe signaling	*NIGT1.3*	GARP-type transcription factor, represses NRT1.1 under Fe deficiency	Activation	[58]
ABA signaling	*ABI5*	ABA-insensitive 5, promotes root-to-shoot Fe translocation	Repression	[58]
Developmental regulator	*TAR2*	Tryptophan aminotransferase, regulates auxin synthesis	Activation	[25]
Symbiotic regulator	*CRE1*	Cytokinin receptor, regulates nodulation	Repression (via NIN)	[33]

**Table 4:** Upstream regulators and interacting partners of NLP proteins.

Interactor	Interacting Partner	Protein Type	Signal/Context	Functional Outcome	References
Upstream kinases	CPK10/30/32	Calcium-dependent protein kinase	Nitrate	Phosphorylates NLP7 at Ser205, promotes nuclear retention and activation	[15]
	CPK28	Calcium-dependent protein kinase	Cold	Phosphorylates NLP7 at Ser783/793/807/808, triggers nuclear import	[20]
	SnRK1(KIN10)	Energy sensor kinase	Carbon deficiency	Phosphorylates NLP7 at Ser125/Ser306, promotes cytoplasmic degradation and inhibits signaling	[53]
Ubiquitin Ligase	HOS1	E3 Ubiquitin ligase	Nitrate	Ubiquitinates KIN10 for degradation, releases NLP7 from inhibitory phosphorylation	[59]
PB1-mediated interactors	NLP6	NLP family transcription factor	Nitrate	Heterodimerizes with NLP7 via PB1 domain, forms higher-order complexes	[32]
	TCP20	TCP transcription factor	Nitrate	Interacts with NLP7 PB1 domain, co-regulates nitrate-responsive genes	[27]
	NIN	RWP-RK transcription factor	Symbiosis (legumes)	Interacts with NLP homologs via PB1 domain, regulates nodulation	[33]
Upstream repressors	SPL9/SPL23	SBP-box transcription factors	Nitrogen-rich conditions	Repress NLP7 expression via miR156-SPL module	[26]
SUMOylation machinery	SUMO E3 ligases	SUMO conjugation enzymes	Nitrate (late phase)	SUMOylate NLP proteins, reduce or enhance transcriptional activity (feedback)	[54,60]

## Data Availability

No new data were created or analyzed in this study. Data sharing is not applicable to this article.

## Supplementary Materials

The following supporting information can be downloaded at: https://www.mdpi.com/article/10.3390/plants15213284/s1, Figure S1: STRING-based interaction network of NLP7 and its associated partners in *Arabidopsis thaliana*.

## Abbreviations

The following abbreviations are used in this manuscript:

NO_3_^−^	Nitrate
NLPs	NIN-like proteins
NIN	Nodule Inception
HSFA2	Heat shock transcription factor A2
TAR2	Tryptophan aminotransferase related 2
NRE	Nitrate-responsive cis-element
NIGT1.4	Nitrate-inducible GARP-type transcriptional repressor 1.4
HSP	Heat shock protein
HSA32	Heat stress associated 32
PB1	Phox and Bem1
ABA	Abscisic acid
CRE1	Cytokinin response 1
