# Supplementary material for: From Nitrate Perception to Environmental Adaptation: Functional Elucidation and Regulatory Networks of the Plant NLP Family
Source: Plants (Basel). 2026 Oct 24;15(21):3284. doi: 10.3390/plants15213284 (PMC13766177; doi:10.3390/plants15213284)
Supplement: Supplementary file 1 [file plants-15-03284-s001.zip › plants-4587119-supplementary.pdf]

To systematically visualize the protein partnerships of NLP7, we constructed a STRING-based interaction network using *Arabidopsis thaliana* as the reference organism (Figure S1). The network substantiates the central role of NLP7 in integrating nitrate signaling with stress and developmental pathways.

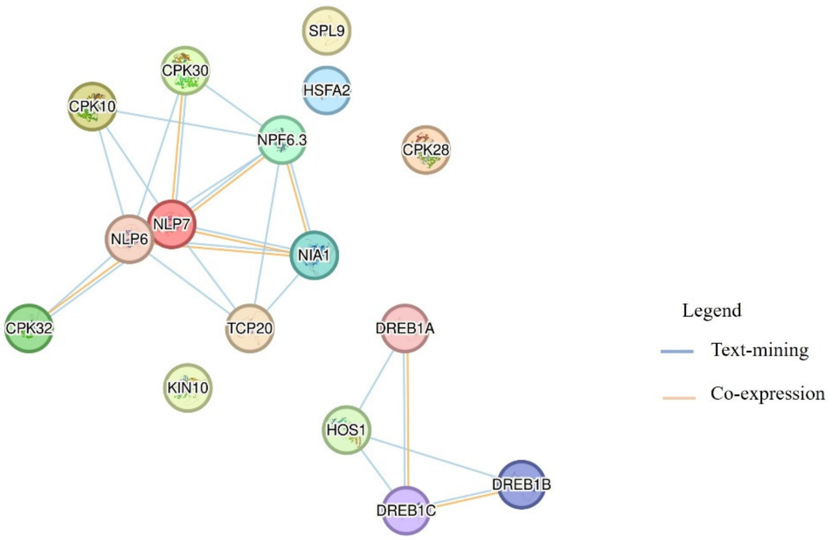

Figure S1. STRING-based protein–protein interaction network of NLP7 and its associated partners in *Arabidopsis thaliana*. The network was generated using the STRING database (version 12.5) with a high confidence score (0.700). Nodes represent proteins, and edges represent functional and physical associations. Edge colors indicate the type of evidence: orange (co-expression) and light blue (text-mining). The network highlights NLP7's connections to nitrate signaling components (NRT1.1/NPF6.3, NIA1), kinases (CPK10, CPK30, CPK32), PB1-mediated interactors (NLP6, TCP20), and downstream transcription factors (DREB1A/B/C/CBFs). Note: *KIN10* and *CPK28* appear as isolated nodes because STRING does not capture transient kinase-substrate phosphorylation events as high-confidence interactions. Similarly, *SPL9* and *HSFA2* appear as isolated nodes because STRING does not capture transcriptional regulatory relationships (transcription factor–promoter binding). The roles of these proteins in regulating *NLP7* (downstream) or being regulated by *NLP7* (upstream) are discussed in the text and summarized in Tables 3 and 4.
